# Setup uncertainties and the optimal imaging schedule in the prone position whole breast radiotherapy

**DOI:** 10.1186/s13014-019-1282-4

**Published:** 2019-05-09

**Authors:** Shengyu Yao, Yin Zhang, Ke Nie, Bo Liu, Bruce G. Haffty, Nisha Ohri, Ning J. Yue

**Affiliations:** 1 Department of Radiation Oncology, Rutgers Cancer Institute of New Jersey and Rutgers Robert Wood Johnson Medical School, Rutgers, The State University of New Jersey, New Brunswick, New Jersey USA; 2 Department of Radiation Oncology, Shanghai General Hospital, Shanghai, China

**Keywords:** Prone-positioned breast, Image guidance frequency, Setup error, LAD

## Abstract

**Background:**

To investigate the setup uncertainties and to establish an optimal imaging schedule for the prone-positioned whole breast radiotherapy.

**Methods:**

Twenty prone-positioned breast patients treated with tangential fields from 2015 to 2017 were retrospectively enrolled in this study. The prescription dose for the whole breast treatment was 266 cGy × 16 for all of the patients and the treatments were delivered with the SSD setup technique. At every fraction of treatment, patient was firstly set up based on the body localization tattoos. MV portal imaging was then taken to confirm the setup; if discrepancy (> 3 mm) was found between the portal images and corresponding plan images, the patient positioning was adjusted accordingly with couch movement. Based on the information acquired from the daily tattoo and portal imaging setup, three sets of data, named as weekly imaging guidance (WIG), no daily imaging guidance (NIG), and initial 3 days then weekly imaging guidance (3 + WIG) were sampled, constructed, and analyzed in reference to the benchmark of the daily imaging guidance (DIG). We compared the setup uncertainties, target coverage (D_95_, D_max_), V_5_ of the ipsilateral lung, the mean dose of heart, the mean and max dose of the left-anterior-descending coronary artery (LAD) among the 4 imaging guidance (IG) schedules.

**Results:**

Relative to the daily imaging guidance (IG) benchmark, the NIG schedule led to the largest residual setup uncertainties; the uncertainties were similar for the WIG and 3 + WIG schedules. Little variations were observed for D_95_ of the target among NIG, DIG and WIG. The target D_max_ also exhibited little changes among all the IG schedules. While V_5_ of the ipsilateral lung changed very little among all 4 schedules, the percent change of the mean heart dose was more pronounced; but its absolute values were still within the tolerance. However, for the left-sided breast patients, the LAD dose could be significantly impacted by the imaging schedules and could potentially exceed its tolerance criteria in some patients if NIG, WIG and 3 + WIG schedules were used.

**Conclusions:**

For left-side whole breast treatment in the prone position using the SSD treatment technique, the daily imaging guidance can ensure dosimetric coverage of the target as well as preventing critical organs, especially LAD, from receiving unacceptable levels of dose. For right-sided whole breast treatment in the prone position, the weekly imaging setup guidance appears to be the optimal choice.

## Background

Breast cancer is one of the most common cancers in women and radiotherapy after breast-conserving surgery is an effective way to reduce the risk of recurrence [1–3]. Conventionally, the whole breast radiation treatment is delivered with patient in the supine position. On the other hand, there is evidence to show that some patients with large breast may benefit from radiation treatment delivered in the prone position in terms of reduced doses to lung and heart [4–6]. However, since it is more difficult to immobilize patient in the prone position, it is more challenging to reproduce the patient setup positioning based on the tattoo location alone [7, 8]. Thus, daily MV portal imaging guidance is considered to be needed for the accuracy and reproducibility of treatment setup, at the expense of added imaging doses to patient [9]. An alternative approach is to increase the beam margin (> 7 mm) to ensure the target coverage [10], which also leads to inclusion of additional normal tissues into the treatment fields.

In the conventional whole breast radiation treatment in the supine position, the current common practice for treatment setup is to position patient based on the body tattoos every day, in conjunction with a weekly portal imaging schedule. This approach takes advantage of setup accuracy based on the imaging guidance while not adding extravagant imaging doses to patient. The question is whether there exists an optimal imaging guidance schedule for the prone-positioned whole breast treatment, with which the imaging dose is minimized without compromising the treatment quality. This study was undertaken to answer the question by evaluating the setup uncertainties and the dosimetric impacts of 4 different imaging guidance schedules: daily portal MV imaging guidance (DIG), weekly portal MV imaging guidance (WIG), no imaging guidance (NIG), and a special imaging guidance schedule in which the first three fractions of treatment were MV portal imaging guided then the averaged setup position was used as the baseline for the subsequent treatments under a weekly imaging guidance (3 + WIG). Among the four imaging guidance schedules, DIG was taken as the reference benchmark.

## Methods

Twenty patients who received the prone-positioned whole breast treatments from 2015 to 2017 in our department were retrospectively enrolled in the current study. In our department, patients are considered for the prone-positioned whole breast treatment based on their anatomy (e.g, large/pendulous breasts which would create an inframammary skin fold in the supine position), heart exposure (e.g, left sided and patient who cannot tolerate breath hold or breath hold is suboptimal), and sometime patient’s own request. Patients who require nodal irradiation are normally not treated in the prone position. Of the 20 patients, 11 underwent left-sided breast treatment and 9 right-sided breast treatment.

### Simulation and treatment planning

**Fig. 1:**
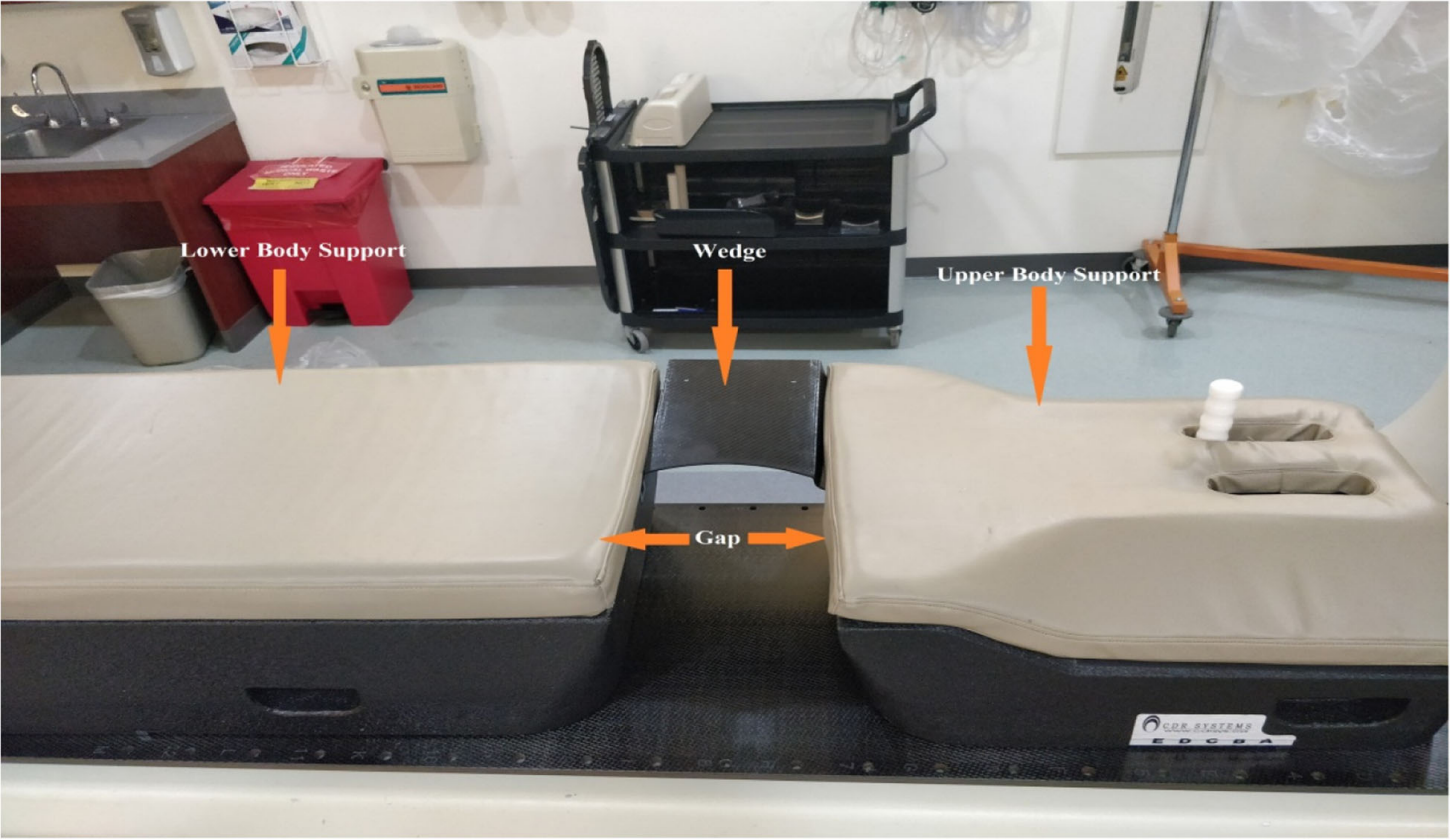
The prone position breast board (Lower Body Support, Wedge, Upper Body Support)

The simulation CT images were acquired with a GE LightSpeed16 slice CT scanner (GE Healthcare, Milwaukee, WI). The patients were first positioned on a prone breast board (Fig. 1, CDR Systems, Calgary, Canada) on the CT table with the breast lying in the middle of the board gap. The contralateral breast was pulled away and supported by the wedge of the board to minimize the exposure to the eventually planed treatment fields. The wedge can be positioned at left or right, depending on which side of breast is to be treated. Markers were placed at the middle of the hanging breast according to the laser both in the left and right. Normally, patient is tattooed at five locations: one medially, one laterally, and three on the back of patient. Free breathing CT scan was then conducted with 2.5 mm slice thickness. The CT scan was transferred to the treatment planning system of the department (Eclipse TPS, V11, Varian Medical Systems, Palo Alto, CA) for the whole breast treatment planning. Lung and heart were contoured on the CT scans as organs at risk (OAR). All of the patients were planned and treated with a fraction scheme of 266 cGy × 16 fractions for the whole breast using two opposed tangential photon beams of 6 or 10 MV. The field in field technique is generally used to achieve the relatively uniform dose distribution inside breast. This study did not include any cone down fractions. To avoid potential collision issues, the SSD (SSD = 100 cm) treatment technique was used. For the left-sided breast patients, since the irradiation of the left-anterior-descending coronary artery (LAD) may cause cardiovascular side effect [11, 12], LAD was also contoured as an organ at risk according to the published criteria [13–15] and was expanded by a 1 cm margin to account for respiratory and cardiac motion [16, 17].

**Fig. 2:**
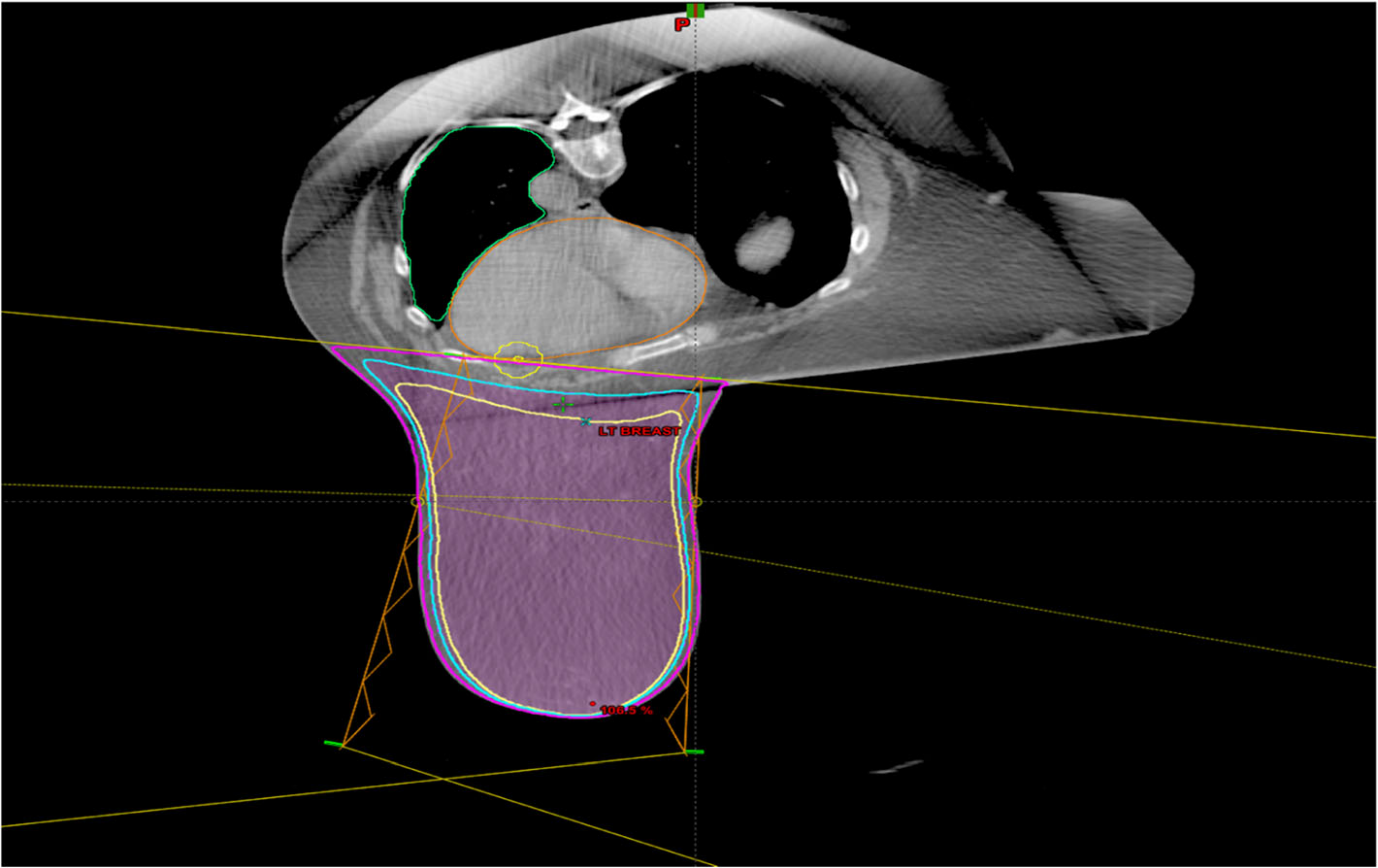
A typical treatment plan of the prone position whole breast treatment. The yellow, cyan and pink line in breast is the dose line (100, 95, 70%); The straight yellow line is the beam line

For the target coverage, the treatment plans were evaluated based on the dose distribution of 95% isodose line of the prescription dose and the maximum dose (Dmax) inside breast. To estimate the impact of setup uncertainties on the target dose coverage, the 95% isodose lines in the plans were converted to structure contours and were evaluated as CTVs in the analysis. Figure 2 shows the dose distribution on a CT slice for a typical plan.

### Treatment setup image acquisition and setup uncertainties

**Fig. 3:**
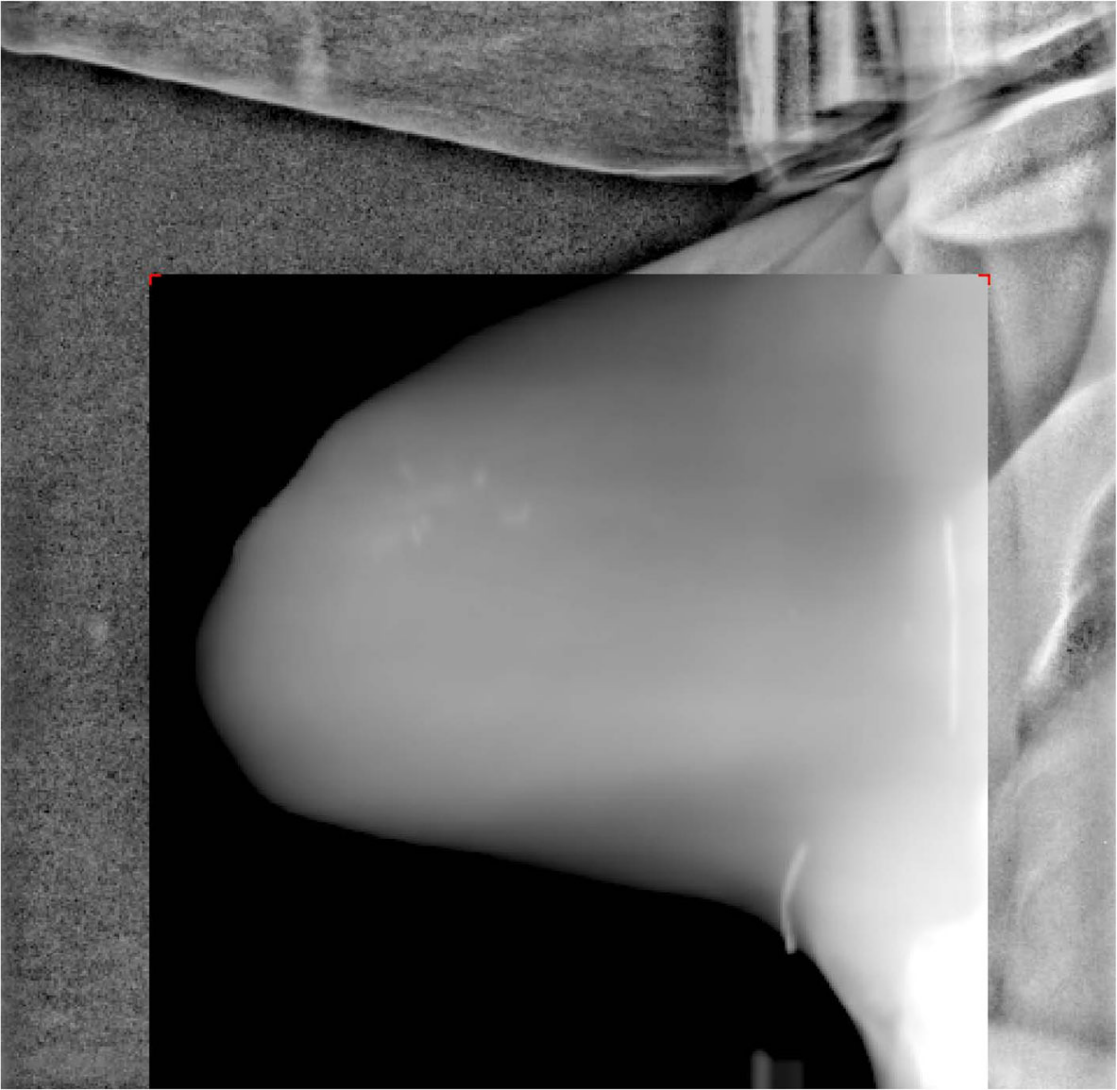
An example of the matched MV portal image with the corresponding DRR

At each fraction of treatment, patient was firstly set up based on the body tattoo locations. The position of the treatment couch was recorded in the record and verify system (RV) (ARIA, Varian Medical Systems, Palo Alto, CA). The MV portal images were then taken as in the same directions of corresponding tangential fields and matched to the corresponding digitally reconstructed radiographs (DRR) for the final treatment positioning (SSD was kept as 100 cm). Figure 3 shows one example of the matched images. This treatment couch position was also recorded in the RV system and was the benchmark in the study. The differences between the tattoo based couch position and the benchmark position were considered the setup uncertainties for no imaging guidance setup. Since the SSD setup technique was used in treatment, only two couch variables were involved in the positioning adjustment: Vertical (Vrt) and Longitudinal (Lng). Of the total 320 (20 × 16) fractions, 319 sets of data were deemed complete, and were collected and analyzed. In the analysis, the systematic uncertainty of an imaging guidance schedule was the mean of all the individual fractional setup uncertainties for that particular schedule and the random uncertainty was the standard deviation.

### Investigated image guidance schedules

Four imaging guidance (IG) schedules were sampled and constructed from the 319 sets of data point: daily IG (DIG), no IG (NIG), weekly IG (WIG), and 3 days+weekly IG (3 + WIG). The setup data for NIG were derived from the recorded treatment couch positions right after the daily body tattoo setup but before the imaging guidance match; DIG was based on the recorded treatment couch positions after the imaging guidance match; WIG was based on the setup of imaging guidance for the first day and the body tattoo setups for the 4 days thereafter and repeated until the end of treatment; 3 + WIG was a specially designed imaging guidance schedule in which the imaging guidance is performed for the first three fractions of treatment and the averaged imaging based setup location is used for the subsequent setup benchmark (if the average setup difference between the tattoo based and imaging based is greater than 3 mm), aided thenafter with weekly IG. All the setup uncertainties were analyzed in reference to the DIG schedule.

## Statistical analysis

The setup uncertainties were analyzed for NIG, WIG and 3 + WIG schedules, relative to DIG, following the approach by Adamson et al. [18]. The systematic (Σ) and random (σ) errors were respectively computed for the three schedules [18, 19].

The dosimetric impacts of the setup uncertainties were investigated for the three imaging schedules, respectively, with the assumption that the patient body shape, size, and anatomy did not experience significant changes throughout the treatment course, and the assumption that DIG based treatment would deliver the plan dose distributions. The investigated dosimetric impacts included the changes of target coverage, the doses to ipsilateral lung, and the heart doses for the left-sided breast patients. The investigated dosimetric parameters included the CTV (95% of prescription coverage volume) coverage and the maximum dose inside the breast volume, the mean dose to heart and the mean and maximum doses to LAD. The dose calcuations were conducted and analyzed for each of the recorded couch position. The impact to V5 of ipsilateral lung was also evaluated. SPSS22 was used for data analysis, *p*-value < 0.05 was considered as statistically significant.

## Results

### Setup uncertainty magnitude and frequency

**Fig. 4:**
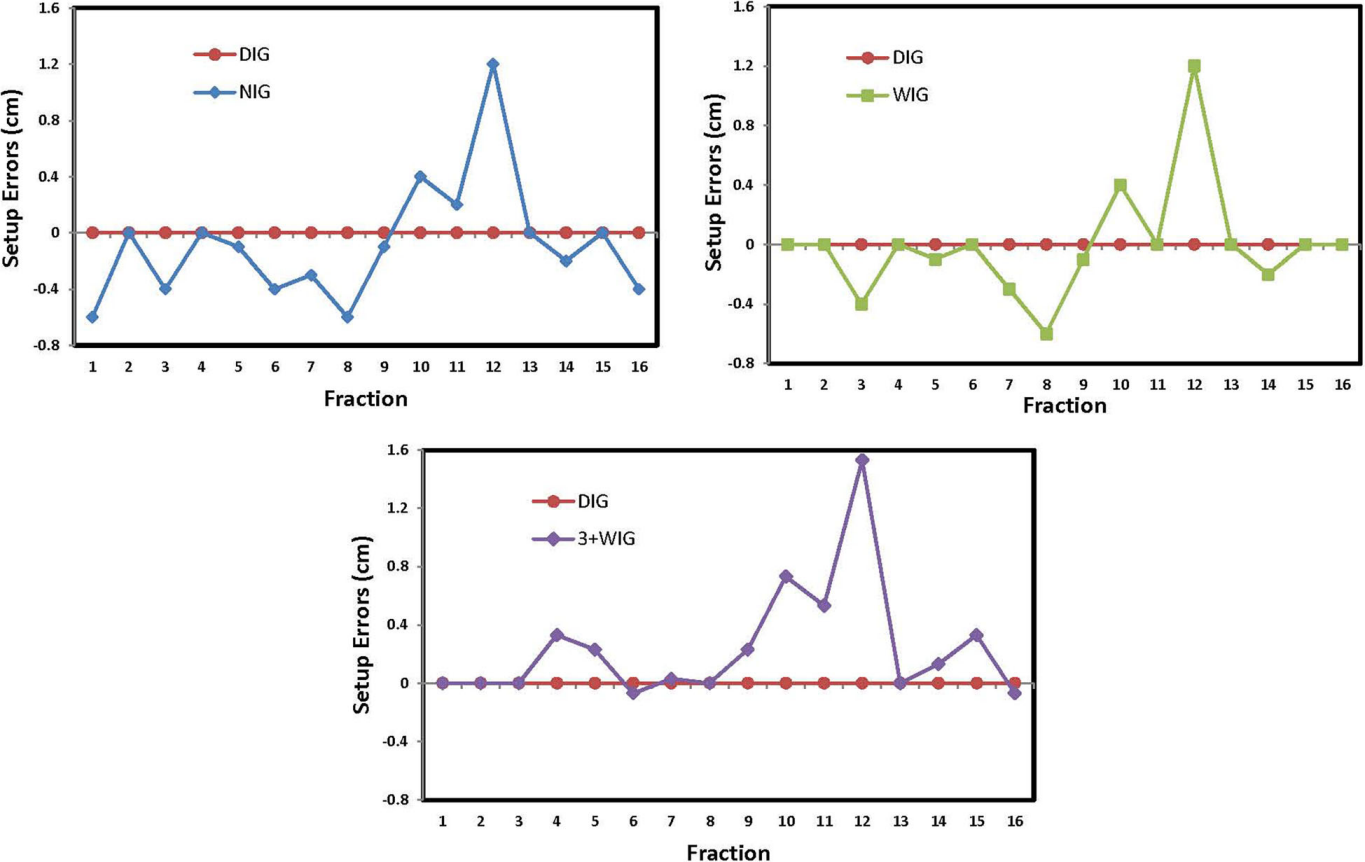
Residual setup errors in the vertical direction of the 4 IG schedules for one of the patients

Table 1 presents the setup uncertainty distributions for NIG, WIG and 3 + WIG schedules. The distributions were categorized into four groups: > 3 mm, > 5 mm, > 7 mm, and > 10 mm. The maximum setup uncertainties are also presented in the table. It is evident that the NIG schedule led to the most frequent setup uncertainties in all of the 3 groups of error magnitude, followed by WIG and 3 + WIG. Although 3 + WIG appeared to improve the setup uncertainty, its differences from WIG were not significant. With all three imaging guidance schedules, the maximum setup certainties were observed to be similar, between about 12.0 mm and 15.3 mm. Figure 4 illustrates the residual setup uncertainties in the vertical direction of the three schedules over the course of treatment for one of the patients. The reference DIG data are also presented in Fig. 4, although they are all zero by definition.

**Table 1:** Residual setup uncertainties of the 3 IG schedules

	Direction	> 3 mm	> 5 mm	> 7 mm	> 10 mm	Max (mm)
NIG	Vrt	47.3%	24.1%	13.2%	5.6%	14.0
	Lng	37.0%	18.2%	8.2%	2.2%	13.8
WIG	Vrt	34.6%	17.8%	9.0%	2.8%	14.0
	Lng	28.7%	14.6%	6.9%	2.2%	13.8
3 + WIG	Vrt	33.0%	16.8%	6.2%	1.4%	15.3
	Lng	28.0%	10.0%	5.3%	1.4%	12.0

*Abbreviations*: *IG* image guidance, *Vrt* Vertical, *Lng* Longitudinal

### Systematic and random setup errors

The systematic (Σ) and random setup (σ) uncertainties were analyzed and computed for NIG, WIG and 3 + WIG. The results are shown in Table 2. It is apparent that the NIG schedule led to the largest systematic and random setup uncertainties. The WIG and 3 + WIG led to smaller uncertainties, with 3 + WIG slightly better than WIG.

**Table 2:** Systematic and random setup uncertainties for the 3 IG schedules

IG frequency	Vrt (mm)		Lng (mm)	
	Σ	σ	Σ	σ
NIG	2.1	3.7	2.8	3.3
WIG	1.8	3.4	2.2	3.2
3 + WIG	1.7	3.4	1.5	2.9

*Abbreviations*: *Σ* systematic error, *σ* random error, *IG* image guidance, *Vrt* Vertical, *Lng* Longitudinal

### Dose changes of OARs

**Fig. 5:**
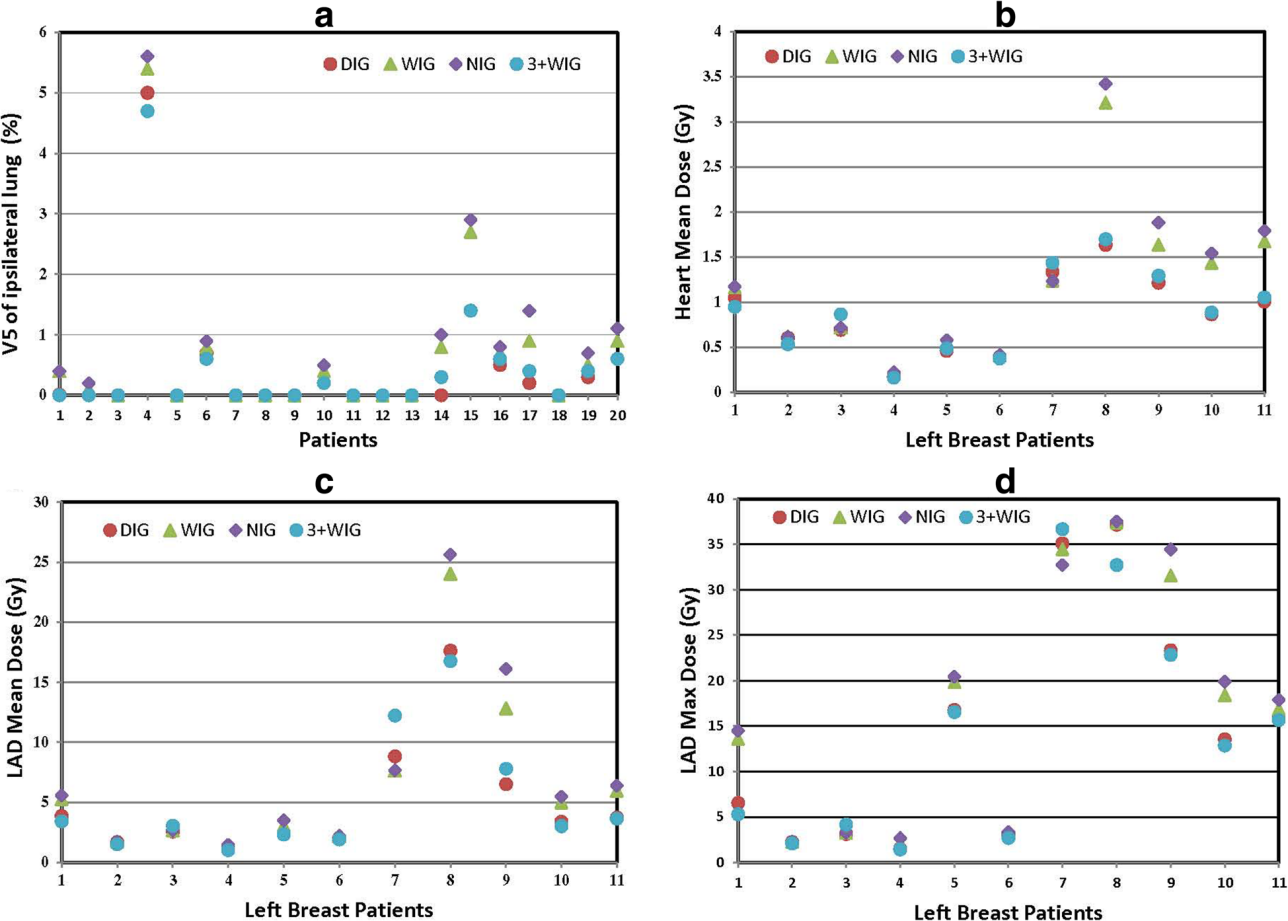
Doses of Organs at Risk for the 4 IG schedules. **a** V5 values of Ipsilateral Lung for all the 20 patients. **b** Heart mean doses. **c** LAD mean doses. **d** LAD maximum doses. **b**, (**c**) and (**d**) are for the 11 left-sided breast patients

The setup uncertainties might very likely cause changes of dose distributions in treatment, not only to the target coverage but also the adjacent organs at risk. Since no CBCT scans were acquired at treatment, the dosimetric impacts were investigated with the assumption that patient body contours and anatomy did not change significantly throughout the course of treatment. The dose distributions for each of the fractions were computed based on the corresponding CT scan with treatment beam repositioned based on the corresponding setup uncertainties. Table 3 shows the statistical comparison of the OAR doses between the 3 less than daily IGs and the DIG. In the table, the values of V5 of the ipsilateral lung were averaged over all 20 patients while the doses to heart and LAD were averaged over the 11 left-sided breast patients. The percent mean dose of OARs increased significantly in WIG and NIG, and the NIG had the largest dose increase. The OAR doses of 3 + WIG increased little, but the maximum dose of LAD decreased. However, the mean absolute doses of OARs did not change much among all the imaging guidance schedules. Figure 5a shows the values of V5 (averaged over the 16 fractions) of the ipsilateral lung for the 4 imaging guidance schedules (the values of DIG represent the plan values) for all 20 patients. In general, NIG increased the V5 the most, followed by WIG and 3 + WIG. Figure 5b illustrates the values of the mean heart dose (averaged over the 16 fractions) for the 11 left-sided breast patients. NIG led to noticeable increase of the heart mean dose in 9 of the 11 left-sided patients, followed by WIG (5 patients) and 3 + WIG (2 patients). For 2 of the patients, the 3 + W IG led to lower heart mean dose. Figure 5c and d show the impacts to the LAD mean dose and max dose, respectively, for the 11 left-sided patients. In most of the left-sided patients, LAD mean dose exhibited little changes for all the imaging guidance schedules. However, for 2 patients (patient 8 and 9), the LAD mean doses increased significantly with the NIG and WIG schedules. For patient 8, the LAD mean dose increased by 47% from 17.4 Gy to 25.6 Gy with NIG, and by 38% from 17.4 Gy to 24.0 Gy with WIG; For patient 9, the LAD mean dose increased by 162% from 6.2 Gy to 16.1 Gy with NIG, and by 108% from 6.2 Gy to 12.8 Gy with WIG. Similarly, in general, NIG and WIG led to more increase of the maximum dose to LAD. For patient 9, the LAD maximum dose also increased significantly with NIG and WIG.

**Table 3:** Mean dose comparisons of the organs at risk- WIG、NIG、3 + WIG vs DIG

	DIG	WIG	Change	*p*	NIG	Change	*p*	3 + WIG	Change	*p*
V5-Lung(%) (20 patients)	0.45±	0.68±	51.1%	< 0.05	0.77±	71.1%	< 0.05	0.46±	2.2%	NS
	1.13	1.28			1.34			1.06		
Heart-mean (cGy) (11 left breasts)	85.71±	117.11±	36.6%	NS	123.46±	44.0%	NS	88.52±	3.3%	NS
	44.1	84.35			91.64			47.18		
LAD-mean (cGy) (11 left breasts)	488.13±	651.21±	33.4%	NS	711.98±	45.9%	NS	515.03±	5.5%	NS
	478.78	668.78			739.84			505.05		
LAD-max (cGy) (11 left breasts)	1437.52±	1665.75±	15.9%	< 0.05	1717.79±	19.5%	< 0.05	1352.44±	−5.9%	NS
	1290.6	1328.4			1339.1			1173.77		

*Abbreviations*: NS-not significant. Paired t test, significant at *p* < 0.05

### Dose change of tumor coverage

It is conceivable that the target coverage would also be impacted by the setup uncertainties. The impacts were investigated for the CTV (the volume covered by the 95% prescription isodose line in plan) and D_max_ inside breast. Table 4 presents the results. The results show that the dosimetric coverage of the CTV and D_max_ were little impacted by the setup certainties for all the imaging guidance schedules. For all of the 20 patients except for one, the CTV coverage decrease was all less than 3%; none was decreased by more than 5%, and the 3 + WIG led to the largest CTV coverage decrease. Only one patient exhibited an D_max_ increase by more than 1%, none of the patients exhibited an D_max_ increase by more than 3%. The differences were not statistically significant.

**Table 4:** Dosimetric impacts to the target coverage: number of patients with decrease of D95 and increase of Dmax

IG	D95			Dmax	
	> 1%	> 3%	> 5%	> 1%	> 3%
DIG (reference)	0	0	0	0	0
WIG	2	0	0	1	0
NIG	0	0	0	1	0
3 + WIG	9	1	0	1	0

## Discussions

In our study, the setup uncertainties were analyzed in relative to DIG, with the assumption that DIG would produce minimal if not zero setup uncertainty. Although the assumption may be likely valid, it is understood that residual setup uncertainty should still exist depending on the matching skill and judgment of the operator. The residual setup uncertainty may well also be caused by the breathing and other types of physiological motion of patient. As shown in Table 1, the setup uncertainties trended to decrease with the increasing use of imaging guidance, consistent with the findings of Zeidan et al. [20, 21]. Zeidan et al. also concluded that residual setup errors reduce with increasing frequency of IG during the course of external-beam radiotherapy, althougth that study was for head and neck patients treated with helical tomoterapy. However, although the 3 + WIG schedule seemed to reduce the residual uncertainties, on occasional fractions of treatment, it led to larger maximum setup uncertainties (Table 1), similar to the findings of a lung patient study by Higgins et al. [22]. One of the possible explanations is that the systematic correction based on the initial imaging guidance helped reduced the mean error but might cause the error magnitudes of certain fractions larger due to the mean shift applied in the opposite direction. The other possible causes were patient’s physiological motion (e.g., breathing, swelling) and improper breast positioning when patient was lying on the treatment couch.

In our department, the following criteria are generally followed for the initial treatment plan in the prone-positioned whole breast irradiation: 1) the maximum point dose should be lower than 108% of prescription dose, 2) no more than 2 cc of breast volume receive higher than 107% of prescription dose, and 3) no more than 200 cc of breast volume receive higher than 105% of prescription dose, 4) for lung, although in general V_20Gy_ of ipsilateral lung needs to be less than 20%, V_20Gy_ is almost always very low for the prone-positioned whole breast treatment, and 5) the mean heart dose should be lower than 2 Gy. In the study, no violation was found for the breast coverage due to the setup uncertainties; however, for the mean heart dose, violations were indeed found, and LAD max dose was also impacted although it was not separately considered in the initial plan.

The dosimetric impacts to the target coverage and OAR doses by the setup uncertainties were investigated by assuming that the patient body contours and anatomy did not differ significantly from the corresponding plans. This assumption is obviously not completely true since it is almost inevitable that patient anatomy may exhibit certain variations. The assumption may more or less affect the accuracy and precision of the dosimetric results in the study.

For the target coverage, since the open opposed tangential fields were used in treatment, it is not unexpected that the target coverage did not exhibit significant changes among the investigated imaging schedule, even for the treatment without any imaging guidance. Among all imaging guidance schedules, although it appeared that the 3 + WIG led to more patients for the CTV coverage decrease, the decrease magnitude was not significant enough. However, for the OAR doses, the changes were more pronounced. For the lung and heart doses, although the relative changes were significant, the absolute dose changes were relatively small and the changed doses were still below the corresponding criteria limits. For left-sided breast radiotherapy, it has been shown that LAD dose is more relevant to causing cardiovascular diseases [11, 12], while the irradiated heart volume is less likely to cause cardiac mortality [23], which indicates that the increase of LAD dose is possibly more a concern than the increase of heart dose. Our study shows that in all the 3 non DIG imaging guidance schedules, for some individual patients, the LAD mean and maximum doses could exhibit non-negligible increases in some fractions of treatment. The results may indicate that for left-sided breast patients treated in prone position with the SSD setup technique, daily imaging guidance may be warranted despite higher imaging dose (which is usually less than 100 cGy over a course of treatment of 16 fractions using the MV portal imaging technique).

Image guided radiotherapy (IGRT) can improve the accuracy of target positioning, reduce the doses of OARs [24, 25]. However, daily IGRT introduces extra doses to patient. For the MV portal imaging, the extra dose to patient is about 1-5 cGy for two opposed beam imaging [26]. For Linacs equipped with kV imaging device (e.g. OBI), the use of 2D kV imaging may significantly reduce the imaging doses to patient, which is about 1–2% of the MV imaging dose [27]. However, for the current OBI systems equipped to the Linacs, the 2D kV imaging can only be used for the verification of the isocenter; the 3D CBCT is of course superior in terms of positioning verification but leads to comparable or higher doses than the MV imaging approach. Additionally, since the breast is not rigid, simple verification of isocenter, as what the 2D kV IGRT can offer, may not be adequate for ensuring target coverage. Therefore, the MV portal imaging is still a good choice for the whole breast treatment setup guidance.

Recently, a paper [28] was published on the similar topic as in our study. In that paper, the group used kV based IGRT to explore the optimized imaging schedule for prone positioned breast external beam radiothearpy. In our study, the data from the MV IGRT portal imaging, which allows more direct anatomy alignment inside the beam portals, was analyzed for the optimal imaging schedule. To the best of our knowledge, our study was the first to draw a conclusion on the optimal imaging schedule for the prone breast radiotherapy using the MV portal imaging IGRT techinique.

In this study, the MV setup imaging was taken along the direction of corresponding tangential treatment fields. Since the tangential treatment fields are normally arranged to spare as much dose as possible to normal organs, imaging along the tangential fields does not expose additional normal organs with radiation, unless a larger imaging field is used. On the other hand, the orthogonal field approach would inevitably expose normal organs beyond the treatment fields.

According to the results of this study, it appears that even the NIG schedule would not lead to underdosing the target volume. However, since all the imaging guidance schedules of the study were sampled and constructed from the treatments with the DIG, the NIG data may be biased since the initial tattoo based setup data at every fraction of treatment might have been influenced by the daily imaging guided setup, especially for those after the very first treatment. Therefore, the WIG schedule seems to be the optimal imaging guidance schedule, especially for right-sided whole breast treatment in the prone position.

In some institutions, the breast patient setup is based on the patient breast surface matching. Therefore, the conclusion drawn from this study may need to be evaluated before applying to the clinical treatments.

The results of the current study were based on the treatments with the SSD setup. The findings of this study need to be validated for treatments using the SAD setup.

## Conclusions

For right-sided whole breast radiation treatment in the prone position, the weekly imaging guidance with the MV portal imaging system appears to be the optimal choice. The schedule does not compromise the target coverage neither the doses to OARs while not adding excessive imaging doses. It also provides a weekly imaging guided setup check to avoid any potentially significant miss which may happen without any imaging guidance throughout a course of treatment. For left-sided whole breast radiation treatment in the prone position, although the weekly imaging guidance schedule does not compromise the target coverage, it may leave room for occasional significant increase of LAD dose. Thus, the daily imaging guidance schedule appears to be optimal for the left-sided breast to prevent any chance of significant increase of LAD dose.

## Abbreviations

3+WIG: initial 3 days then weekly imaging guidance
CTV: Clinical target volume
DIG: Daily imaging guidance
IG: Imaging guidance
LAD: the left-anterior-descending coronary artery
NIG: No daily imaging guidance
OAR: Organ at risk
OBI: On board imaing
SAD: Source axis distance
SSD: Source surface distance
WIG: Weekly imaging guidance
